# 

**Published:** 1893-04-22

**Authors:** 


**OUR NEW OFFICES.**
428, Strand, W.C., will henceforth be the Office of this Journal.

**Notices of Births, Deaths, and Marriages,** are inserted in this column at a charge of 2s. 6d. for an announcement not exceeding four lines.

### NOTICE TO CORRESPONDENTS.

All MS., Letters, Books for Review, and other matters intended for the Editor should be addressed The Editor, The Lodge, Porchester Square, London, W.
The Editor cannot undertake to return rejected M.S., even when accompanied by stamped directed envelope.

**Notice to Advertisers and Subscribers.**

All Advertisements, Orders for Copies of The Hospital, and Business Communications should be addressed to The Publisher, *not to the Editor*, The Hospital, 428, Strand, W.C.

The Cost of Subscription, post free, is as follows:—Per week, 2½d.; per quarter, 2s. 6d.; per half-year, 5s.; per annum, 8s. 6d. Foreign:—Per annum, 10s. 8d.; payable, in all cases, in advance.

**"Burdett's Hospital Annual," 1893.**

Fourth year of publication. 700 pp. Boards, gilt lettering. A most complete guide to British, Colonial, Indian, and American Hospitals, Dispensaries, Nursing and Convalescent Institutions, and Asylums. Price 5s. Published by The Scientific Press (Limited), 428, Strand, W.C.

**NOTICE.—The Index for Vol. XII. (ending September 1892) is on sale at the Office of this Journal, price 2½d., post free.**

### SCRAPS AND GLEANINGS.

The King of Italy has consented to open the Medical Congress at Rome on September 24 h.

The *New York Times*, which has been sold to a syndicate, realised 2,000,000 dollars at its sale.

The King of Denmark, who is the eldest crowned head in Europe, completed his seventy-fifth year at the beginning of April.

We hear from Vienna that Baron Albert Rothschild has given half a million florins for the erection of a cancer hospital at Vienna.

New York City can boast of an hotel which is nearly a quarter of a mile in length, the height of which, we are glad to say, is not quite in proportion.

Persons suffering from a slight form of deafness have found benefit to their hearing in applying an ordinary Japanese fan as an audiphone, or dentaphone.

Second class railway carriages are to be abolished on the Cambrian Railway after May 1st. Reductions in first-class fares are notified to meet this alteration.

At the conflagration which occurred at the Allan Bradley Distillery, Louisville, 12,000 barrels of whisky were consumed in the flames, at the estimated cost of 600,000 dols.

A bill has lately been introduced into the Missouri Legislature favouring the establishment of Hospitals for the sick and injured employés of the railroads of the State.

Thomas Hart, residing in Australia, has the nearest claim to being a living relative of Shakespeare, he being eight in descent from Joan, who was William Shakespeare's sister.

The trade of bootblacking in public thoroughfares has been entered into by female workers. At Toulon many women gain a livelihood in this manner, uniformed in a neat cap and apron.

A Bill has been introduced into the Legislature of Nebraska which provides for the erection and maintenance of a Patho-Biological Laboratory for original investigation of infectious diseases.

Extensive forest fires have occurred lately in the heart of the Pine district at North Carolina. Several turpentine plantations have been ruined, also houses destroyed, and large quantities of resin.

The French Minister of Public Instruction proposes the creation of a new medical degree superior to the ordinary one of M.D. Probably the degree will take the name of Doctor of Medical Science.

The Liberator Relief Fund reports that the subscriptions amount now to £17,000. The Duchess of Albany, in a letter from Cannes, has expressed great sympathy with the sufferers who have lost their money in this investment.

Duke Charles Theodore of Bavaria completed his two-thousandth operation for cataract at his private hospital at Munich this month. The Duke, who is a member of the Bavarian Family, has operated 1,000 times on cataract during the last four years.

The Police Seaside Home at West Brighton lately received the Prime Minister and his wife as visitors, who farther inspected the new home, which is being erected by voluntary subscriptions at a cost of £8,000, and is intended for the benefit of police from all parts of England.

The Provident Surgical Appliance Society for the Relief of the Crippled Poor is doing a most needful and necessary work. The number of limbs and instruments thus distributed free through "letters" of annual subscribers since the foundation of the institute is 63,425.

Mrs. J. Roberts, widow of the first President of the Republic of Liberia, on the west coast of Africa, is endeavouring to erect a hospital at Monrovia, there being great need of one. Towards this scheme Mrs. Roberts has already secured £600, President Cleveland contributing £10.

The mammoth cheese which has been manufactured as Canada's special exhibit for the World's Fair has been purchased by Mr. T. J. Lipton, the well-known tea and provision dealer. The cheese weighs close on 12 tons, or about 26,880 lbs. This marvel of the dairy was made from the milk of 20,150 cows, and milked by 2,177 dairymaids.

For the Book World of Medicine and Science, and Student of Medicine, and Medical Appointments, see pages VII., IX., and X., overleaf.

### FIRST IMPRESSIONS OF BOOKS RECEIVED

[It is requested that all books intended to be noticed in this column may be sent to the Editor, at the Office, 428, Strand, W.C., not later than Tuesday evening in each week.]

SCAB HEALING AND ITS APPLICATION IN GENERAL SURGERY. By J. DELPRATT HARRIS, M.R.C.S., Surgeon Devon and Exeter Hospital. H. K. Lewis, 1893.

Mr. Harris is an old pupil of the late Mr. Callender's, and he writes these pamphlets to advocate a line of practice first taught him in the wards of St. Bartholomew's Hospital. Combined with the use of careful antiseptics he employs pads of pinewood sawdust to all wounds, and strongly recommends this dressing as simple, cheap, and efficient. Sawdust and wood wool have long been in use as dressings, and are highly commended by many surgeons.

THE HUNTERIAN ORATION. By THOMAS BRYANT, President of the Royal College of Surgeons of England, Consulting Surgeon to Guy's Hospital. (London: Adland and Son, 1893.)

This address was delivered on the centenary of Hunter's death, and in the presence of the Prince of Wales and the Duke of York. Mr. Bryant speaks of Hunter as a man, as a biologist, and as a surgeon, and by many quotations from Hunter's own writings endeavours to bring out the main characteristics of the man and the lines along which he worked and some of the results he attained. The address is full of interest, as anything dealing with this great man cannot fail to be.

MALIGNANT DISEASE OF THE THROAT AND NOSE. By DAVID NEWMAN, M.D., Laryngologist to the Glasgow Royal Infirmary, Assistant Surgeon to the Western Infirmary, &c. (Edinburgh and London: Young J. Penttand.)

The scope of this brochure is sufficiently indicated by its title. Malignant disease of the asophagus, tonsil, larynx, and nose is described, and the author has incorporated with a careful clinical study of these diseases statistical statements which many readers will find useful. Dr. Newman is already well known as an author; the subject on which he now writes is one to which he has devoted considerable attention, and he has produced a very useful book.

### Medical Vacancies and Appointments.

### VACANCIES.

The following vacancies are announced this week, the amount of salary in each case being given after the name of the institution:

### Resident Medical Officers.

Birmingham General Dispensary, Birmingham. Resident Surgeon for the Highgate Branch: doubly qualified. Salary £150 per annum, with allowance for cab hire, and furnished rooms, fire, lights, and attendance. Applications to Alex. Forrest, Secretary, by May 17th.

Bradford Infirmary and Dispensary, Bradford. Honorary Medical Officer. Applications to the Secretary, by April 24th.

Bradford Infirmary. Dispensary Surgeon, doubly qualified, unmarried. Salary £100 per annum, with board. Applications endorsed "Dispensary Surgeon," to William Marr, Secretary, by April 24th.

Chelsea Hospital for Women, Fulham Road, S.W. Clinical Assistant. Fee, three guineas for three months. Applications to the Secretary.

General Hospital, Birmingham. Assistant Obstetric Officer. Honorarium, £100 per annum. Resident Surgical Officer. Salary £130 per annum, with residence, board, and washing. Applications to the House Governor, by April 29th.

General Hospital, Birmingham.—Resident Surgical Officer and Honorary Assistant Obstetric Officer. Salary, Resident Surgical Officer, £130, with board and lodging; Assistant Obstetric Officer, honorary. Applications must be received by April 29th, to House Governor. Election take place on May 5th, at Twelve noon.

General Infirmary, at Leeds. Three House Surgeons. Two for a period of twelve months and one for six months. Board, lodging, and washing provided. Applications to Mr. Littlewood, Secretary to the Faculty, General Infirmary, Leeds, by April 27th.

Great Northern Central Hospital, Holloway Road, N. House Physician. Salary £60 per annum, with board and lodging. Applications to the Secretary by April 24th.

Hospital for Sick Children, Great Ormond Street, W.C. House Surgeon, unmarried, appointment for six months. Salary £25 and board and residence. Surgical Registrar and Anæsthetist. Appointment for one year. Honorarium, £40. Applications to the Secretary by April 25th.

Inverness District Asylum, Inverness.—Assistant Medical Officer, under 30 years of age, doubly qualified. Salary to commence, £100 per annum, with board, apartments, and washing. Applications to Dr. Mackenzie, Medical Superintendent.

Miller Hospital and Royal Kent Dispensary, Greenwich Road, S.E. Junior Resident Medical Officer. Salary £30 per annum, with board, attendance, and washing. Applications to the Honorary Secretary by April 22nd.

North-West London Hospital, Kentish Town Road, N.W.—Resident Assistant Medical Officer, and Assistant Resident Medical Officer. Salary of £50 attaches to the senior post. Appointments for six months. Applications to Alfred Craske, Secretary, by April 25th.

St. Pancras and Northern Dispensary, 126, Euston Road. Resident Medical Officer; doubly qualified. Salary £105 per annum, with residence and attendance. Applications to the Honorary Secretary, H. P. Bodkin, 23, Gordon Street, Gordon Square, W.C., by April 20th.

The Coppice, Nottingham. Assistant Medical Officer; unmarried, not more than 28 years of age. Salary £120 per annum, with furnished apartments, board, washing, and attendance. Applications to Dr. Tate, at the Asylum, by April 24th.

### Non-Resident Medical Officers.

City of London Hospital for Diseases of the Chest, Victoria Park, E. Pathologist. Salary 100 guineas per annum. Applications to the Secretary, at the office, 24, Finsbury Circus, E.C., by April 20th.

Dunshaughlin Union, Dunboyne Dispensary. Medical Officer. Salary £110 per annum, with £15 as Medical Officer of Health, exclusive of registration and vaccination fees. Applications to Mr. Lawrence Ward, Honorary Secretary, The Grove, Clones, co. Meath. Election on April 28th.

Hastings, St. Leonards, and East Sussex Hospital, Hastings. Third Assistant Surgeon. Applications to the Secretary, by April 22nd.

New Hospital for Women, Euston Road, N.W.—Fully qualified medical woman as Clinical Assistant for In-Patient Department. Appointment for six months. Applications to the Secretary, by April 28th.

### MEDICAL APPOINTMENTS.

### THE GENERAL HOSPITAL, BIRMINGHAM.

ASSISTANT OBSTETRIC OFFICER.

RESIDENT SURGICAL OFFICER.

### BIRMINGHAM GENERAL DISPENSARY.

RESIDENT SURGEON WANTED for the Highgate Branch.

Candidates must be registered and possess both a Medical and a Surgical qualification.

Applications, together with ORIGINAL TESTIMONIALS AND CERTIFICATES OF REGISTRATION, must be forwarded to the undersigned on or before WEDNESDAY, May 17th.

Salary £150 per annum, with an allowance for cab hire, and Furnished Rooms, Fire, Lights, and Attendance

(319) ALEX. FORREST, Secretary.

### THE HOSPITAL FOR SICK CHILDREN,

Great Ormond Street, W.C.

